# Football Fans’ Emotions: Uncertainty Against Brand Perception

**DOI:** 10.3389/fpsyg.2020.00659

**Published:** 2020-05-15

**Authors:** Elena Shakina, Thadeu Gasparetto, Angel Barajas

**Affiliations:** National Research University Higher School of Economics, Saint Petersburg, Russia

**Keywords:** brand-teams, Brazilian football, emotions, fans, instrumental variables, professional football, uncertainty of outcome

## Abstract

Football is an industry driven by emotions. Fans experience many different emotions related to their teams. This paper aims to inspect how emotions impact attendance at football matches, examining whether football fans prefer to watch highly competitive matches or matches between good teams with star-players. The paper also considers behavioral and emotional differences of match spectators when brand-teams play away or at home. Importantly, we are also looking for the effects that the expectations of these emotions have on the tickets’ price mechanism. We use data from three seasons of the Brazilian State championship with information on more than 1,100 matches. The OLS estimator with the moderation marginal effects allows for analysis of a brand-team playing with different levels of uncertainty over the outcomes measured by the relative level of the divisions of rivals. We look for the difference between the marginal contribution of the brand-team and the uncertainty of outcomes that might change under some conditions. The analysis is performed later using two subsamples and, finally, we address the problem of endogeneity in price using an instrumental variable. From our results, the main findings are: first, that the price of tickets does not much affect the demand when a brand-team is playing. In case of competitive matches between non-brand-teams, price behavior correlates to the rationality of the demand curve having a negative impact. The fact that price is not relevant for matches with the brand-team comes to corroborate the idea that fans are driven more by emotions than by economic reasoning; second, the phenomena of highly competitive matches does not work when a brand-team is playing against a small one; and third, the effect of a brand-team playing is relatively more important than the uncertainty of outcome. The last two findings mean that the satisfaction of watching star-players or big-teams is stronger than the emotion brought by a competitive match.

## Introduction

Football is an industry driven by emotions. This industry is attracting interest from different arenas and brings not only popularity but also substantial revenues from fans, corporations, and advertisers. Fans experience many different emotions related to their teams, especially on match-days. For instance, the excitement after a remarkable win or the act of hiring a star player drastically differs from the frustration of a championship lost. Therefore, emotions shape fan behavior, influencing participation, engagement, consumption of merchandising, and match attendance. This last point – match attendance – is analyzed in this work. The focus of our paper is to examine fan behavior patterns under specific conditions: whether they prefer to experience the emotion of a high competitive match or to watch well-known teams playing. We also inspect how attendance change when a brand-team – a well-known football club which habitually attracts a higher number of fans – plays at home or away.

We carry out our study on the data of very specific championships at state level. These championships are traditional football tournaments in Brazil and do not have close analogs worldwide. The key distinction of the Brazilian State Championships is that they are structured according to geographic criteria (the Brazilian states and the Federal District are used to organize tournaments) and as a consequence there are matches between very strong brand-teams and local small teams from the same regions. This unique structure – tournaments where only clubs from the same state can play against them – provides an atypical characteristic not found worldwide: matches between clubs with *theoretically* huge sportive differences. Although one would say that it may happen in several Domestic Cups around the world (i.e., *Copa del Rey* in Spain or *FA Cup* in the United Kingdom), it occurs every season in the State Championships. This setting reflects information regarding different kinds of emotions that spectators can experience as the surprise of an underdog beating a ‘big’ brand-team or the expectation generated for a derby match. Evidently, this specific setting may allow us to discover sport-related emotional factors and behavioral biases of spectators which occur only when highly imbalanced matches take place.

Our hypothesis is that match attendance to such events might be affected by a number of specific factors that cannot be observed in conventional football matches. Having said that, we can consider Brazilian state championships as a good laboratory to study specific fans’ preferences and inspect whether the effect of uncertainty in sport outcomes might be weakened by the brand perception.

We use data from three seasons of the Brazilian state championship, selecting three of the most important states: *Minas Gerais*, *Rio de Janeiro*, and *Sao Paulo*. The dataset comprises information on more than 1100 matches, specifically, match attendance, ticket price, scores, venue, day of the matches, teams playing, and their main characteristics: level of division in Brazilian league, previous titles, and brand power.

The econometric strategy follows two steps. The first step utilizes the OLS estimator with the moderation marginal effects. This reduced form specification allows the analysis of brand-team playing with different levels of uncertainty over outcomes measured by relative level of the divisions of opponents. We look for the difference between the marginal contribution of the brand-team and uncertainty of outcomes that might change under some conditions. The second step treats separately two cases of matches with brand-teams and those which involve only non-brand-teams. By using instrumental variables, this step allows for going deeper into the price mechanism affected by irrational emotions of spectators, which may create distortions for normal demand function.

The remainder of this paper is organized in a conventional manner presenting both state of the art sports economic and sports phycology literature. The discovered gap in the literature is studied on specific data which presumably track irrational attitudes of football fans when it comes to highly imbalanced matches. The methodological and empirical findings are presented in the Research Design and Results sections respectively.

## Literature Review

Previous literature addresses emotional elements in the behavior of football fans. Funk et al. (2000) offer a broad review regarding the attitudes of sport fans. The authors discuss the importance of social, cognitive, and psychological elements (i.e., experiences, preferences, identification, etc.) in the involvement of fans in sports. Vallerand et al. (2008) examines passion among football fans, showing that harmonious passion is associated with some positive experiences like identity as a club fan, celebration on the streets, and life satisfaction. On the other hand, they also identify that an obsessive passion would drive maladaptive behaviors by football fans. Martin et al. (2008) offer evidence that emotionally based satisfaction (i.e., happiness, excitement, surprise) positively drives future intention among football fans, increasing their commitment with the club and increasing the likelihood of future attendances. Biscaia et al. (2012) corroborate with these findings, evidencing that pleasant emotions positively influence future behavioral intentions among football fans as well.

Recent research also inspects the relationship between sport fans’ emotions and (sport) product quality and services satisfaction. Foroughi et al. (2016) indicate that anxiety, excitement, and happiness are significantly associated with good players’ performance. They also emphasize that happiness and excitement can positively impact behavioral intentions, driving higher attendances. This last finding is similar to what Madrigal (1995) and Kuenzel and Yassim (2007) earlier observed. Moreover, Yim and Byon (2018) show evidence that sport fans’ emotions differ based on match outcome, where positive sport results are associated with higher game and service satisfaction, leading to positive behavioral intentions, but negative outcomes do not impact service perception. Therefore, they suggest that sport decision-makers have to provide high quality services in order to handle the negative impact of adverse sport outcomes on fans’ emotions.

Some other emotional elements impacting football consumption have been tested recently: suspense, surprise, and shock. Pawlowski et al. (2018) show that perceived suspense strongly encourages fans to watch a football match on TV, while Buraimo et al. (2019) empirically indicate that minute-by-minute broadcast demand is driven by all three of these emotional elements.

Since the seminal papers of Rottenberg (1956) and Neale (1964), the uncertainty of outcome hypothesis (UOH) has been one of the most relevant topics in the economic analysis of sports. However, during this time, empirical research has not been able to provide a clear evidence for the importance of competitive balance (CB) on attendance (Pawlowski and Budzinski, 2013; Sacheti et al., 2014). There is evidence that UOH apply to the Korean Professional Baseball League (Cha et al., 2015), Australian Football League using structural time-series model (Lenten, 2017), Japanese professional soccer (Watanabe, 2012), and Major League Baseball (Soebbing, 2008). However, we cannot say that the increasing balance in soccer brings a risk of fall in consumer demand (Pawlowski, 2013).

Some studies focus on the uncertainty of outcomes considering short-run (match), medium-run (reaching the knock-out stages)m and long-run (season) with different outputs. Hogan et al. (2017), analyzing rugby, found that short-run uncertainty has little effect on attendance, but medium-run has a significant impact. Something similar was found by Jane (2014b) in the NBA where the long-run uncertainty had a significant impact on attendance but not at a game level. On the contrary, Sacheti et al. (2014) discovered that short-run uncertainty in cricket had a significant impact on attendance. In the same line, Lee et al. (2016) sustain that only match uncertainty is statistically significant in Korean Professional Baseball League. Game importance, a measure of game uncertainty, is an influential factor in attendance consistent with the UOH (Lei and Humphreys, 2013). However, Paul et al. (2011) studied hockey attendance in Russia, Sweden, and Finland. They found that in the two first countries, fans present strong preferences for uncertainty of outcome, but this does not happen for Finish fans.

Other studies have found that other factors have a greater influence than the uncertainty of outcome. That is the case of the paper by Serrano et al. (2015). They affirm that match day demand is more related to the expected quality of the contestant teams, approximated by the market value of the players, than the uncertainty of outcome hypothesis. The strength of the home team (Paul et al., 2011; Hogan et al., 2017) and absolute team strength (Sacheti et al., 2014) have a significant effect on attendance. However, Coates and Humphreys (2010) found a weak evidence that attendance increases when a victory of the home team is expected, and a strong evidence that attendance falls when the home team is expected to lose.

There are other factors that may influence the attendance. The league standing effect, earlier described by Neale (1964), also drives football fans attention. Indeed, Andreff and Scelles (2015) empirically confirm its importance, as well as some other papers – analyzing it as competitive intensity – showing its positive effect on both tickets and TV demand (Scelles et al., 2011a, b, 2013a,b; Bond and Addesa, 2019, 2020). Similarly, if the team still is in the fight to win the championship, attendance is higher (Pawlowski and Nalbantis, 2015). Closer wins by the competing teams and larger gaps in points spread between two teams also drive higher attendance (Jane, 2014b). The impact provided by star-players effect is also found. The appearance of star-players increases home and away match attendance (Jane, 2014a), and television audiences as well (Buraimo and Simmons, 2015; Scelles, 2017).

Schreyer et al. (2016) found support to UOH in league, but not in knockout tournament games when analyzing television audiences. However, Alavy et al. (2010) show evidence that in a minute-by-minute analysis uncertainty matters, but the progression of the match drives viewership.

Finally, the participation of a brand-team in the match is also an important element driving higher attendance rates. Gasparetto et al. (2018) define brand-teams as historical clubs with a successful sportive record which attract higher attention from fans – similar terms as big clubs, strong brand clubs, well-known teams, or famous clubs are also used in the literature with identical meaning. Pérez et al. (2015) indicate larger television audiences on matches played by Real Madrid and Barcelona in Spain. Pawlowski and Anders (2012) evidence that a strong brand of the away team increases attendance in the Bundesliga as well. Therefore, the present paper aims to inspect how emotions impact attendance rates, examining whether football fans prefer to attend high competitive matches or matches when good teams are playing.

## Research Design

This study seeks to relatively examine and compare the effects of brand-teams and uncertainty of outcomes, placing special emphasis on emotional factors which can bring distortions to the traditional understanding of rationality of spectators’ incentives. Furthermore, bridging the gap between psychological and economic literature, we expect to explore a price mechanism affected by irrational bias in football fans’ behavior.

To test the uncertainty of outcomes hypothesis against brand-team effect, this paper uses the way of measuring uncertainty of outcome introduced by Gasparetto et al. (2018). This measure of uncertainty of outcome is applied because the analysis includes teams from different divisions in the same competition. As contribution, this work introduces the moderation effect of brand-team on the uncertainty of outcome. For that we have to split the effect of being a brand-team and the effect of uncertainty and then we will compare both effects under different conditions.

The research question in this study is specific as we seek not just to reveal the marginal effect of UO and brands, but to compare these effects applying difference-in-difference technique. Moreover, we challenge the endogenous price effect using the instrumental variable (IV) approach.

**FIGURE 1:**
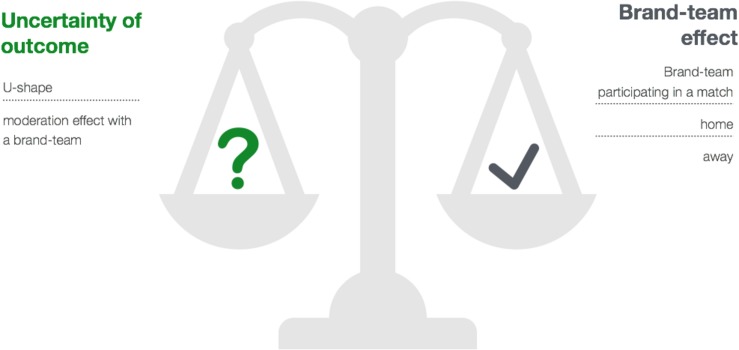
Two-effects-driven attendance of Brazilian State Championship matches.

At it has been pointed out, this study addresses the issue of separating out two effects that both lead to popularity of football matches: uncertainty of outcomes and the effect of strong game provided by well-known brand team. In order to test these phenomena, we design our study in the following steps (Figure 1):

1. Identification of brand-team effect:
  (a) Brand-team are selected according to the following criteria: a club has to have at least 20 State Championship titles and at least one Brazilian League title. Moreover, this club must be one of the *Clube dos 13’* founders.
2. Identification of uncertainty effect:
  (a) Uncertainty is measured by relative difference of league’ tiers in which teams are presented in one match.
  (b) U-shape relationship of uncertainty is introduced in the specification of the model.
3. Comparison of two effects under different conditions:

The difference in moderation effects is measured by the interaction of variables of uncertainty and a dummy of the brand-team playing at home or away.

4. Comparison of a subsample-specific effect of price: with or without brand-teams matches:

The difference in the marginal price effect on different subsamples explicitly demonstrates the endogenous nature of the price. Moreover, the fact that prices of matches with a brand-team is substantially higher, shows that it includes fans’ intentions to attend these matches.

5. Estimation of the exogenous price effect by using instrumental variable technique:

For the instrument, the attendance of the identical match from the previous seasons is taken. It is asserted to validate this instrument that the previous attendance doesn’t have a direct influence on the decision of each particular spectator to attend a current match. Meanwhile, organizers of the matches consider previous attendance as a reference to set price for a current match, since that is a good predictor for their revenues.

Following these steps, we identify the model where attendance of matches (*attendance*) is driven by uncertainty of outcomes in quadratic functional form (*Unc* and *Unc*^2)^, interaction of uncertainty multiplied by a dummy variable of brand-teams (*BT*), controlled by brand-team effect playing home or away and remained control variables (*CV*) such as price for tickets, seasons, level of the match, and region. The specification is introduced in the formula 1.

$$\mathrm{Log}\,{\left(\mathrm{attendance}\right)}_{i\,t}$$

$$=\beta_{0}+\beta_{1}\cdot U\,n\,c_{i\,t}+\beta_{2}\cdot U\,n\,c_{i\,t}^{2}+\beta_{3}\cdot U\,n\,c_{i\,t}\cdot B\,T_{i\,t}+\beta_{4}\cdot B\,T_{i\,t}+\beta_{5}\cdot C\,V_{i\,t}+\varepsilon_{i\,t}$$

We use the OLS estimator to find out and compare two effects. The algorithm of this estimation requires step-by-step performing of examined effects in regression. Four models are presented to demonstrate how effects are changing when an additional significant relationship is introduced. The first model is estimated by ignoring interaction of brand-team effect and uncertainty. The second model covers all possible effects both for brand-team playing home and away. The third model imbeds just brand-team effect and its moderation no matter if they play home or away. And the last model considers just the brand-team playing at home. By exploring a dynamical modification of effects, it enables interpretation of results to draw hypothesis testing.

Notably, the exogeneity of all variables of our interest is driven by rules set up in the Brazilian State Championship. Apparently, these rules are not influenced by expectation of attendance and originate from the idea to bring together all possible combinations of teams from the same Brazilian state based on toss-up. That makes these variables random and, as a result, exogenous for our explained phenomenon. Meanwhile, the key control factor of the model – price for ticket – might be endogenous and requires very precise consideration.

Our research involves classical metrics of matches’ and teams’ diversity. The only one non-conventional metric is related to uncertainty, and is specifically designed for cases studied, although it has been used in Gasparetto et al. (2018). Since different levels of leagues are brought together in matches of the State Championship, that might be a proper base to estimate uncertainty of game outcome. Our assumption is – the greater difference of tiers the lower uncertainty is associated with a corresponded match. Meanwhile, the same high uncertainty is generated when two strong teams from the first division are playing or two non-league teams are opponents in the same game.

The next section introduces the data involved in our analysis and gives a brief statistical overview of their distribution.

## Data

The sample comprises three of the most important Brazilian States Championships: *Carioca*, *Mineiro*, and *Paulista* – in Rio de Janeiro, Minas Gerais, and São Paulo states, respectively. The financial data was gathered from the websites of each State Federation.

The classification of clubs as Brand-team in Brazil was accomplished in two parts. Firstly, we have selected all founder clubs of the *Clube dos 13*, a group created in 1987 by the Brazilian elite teams. The objective of this group was to develop the Brazilian football market (Silva et al., 2014). Moreover, a second criterion was included: Only teams with more than 20 State Championships’ titles and at least one title of the *Campeonato Brasileiro* were selected. Thus, the following clubs are considered brand-teams: Botafogo, Flamengo, Fluminense, and Vasco in the Carioca Championship; Atlético-MG and Cruzeiro in the Mineiro Championship; and Corinthians, Palmeiras, Santos, and São Paulo in the Paulista Championship.

The data set consists of all 1,114 matches over 2013, 2014, and 2015 seasons – 216 of them from Mineiro Championship, 380 from Carioca, and 518 from Paulista. Each State Federation has autonomy in defining the competitive format of its championship. The number of competitors, number of matches, as well as the promotion and relegation system is a decision of the State Federations and of the associated clubs. A detailed description of these championships can be found in Gasparetto et al. (2018).

It is worth pointing out that for the three researched State Championships over the whole period, the average attendance is evidently low in all of the tournaments. Moreover, there is no trend about growing or decreasing – they are slightly similar over the seasons with each championship. On the other hand, the huge standard deviations indicate heterogeneous samples with almost empty stadiums as well as others that are quite crowded – the minimum and maximum values come to confirm it as well.

**FIGURE 2:**
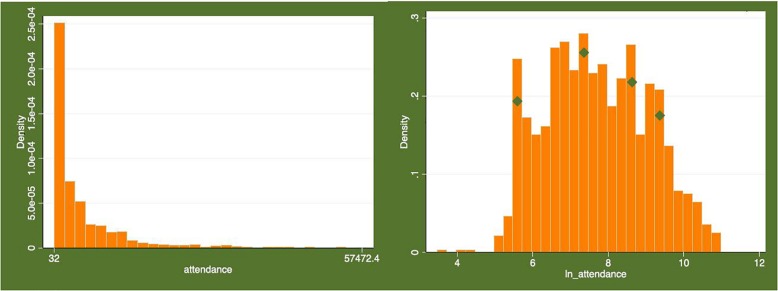
The distribution of attendance on matches in Brazilian State Championship presented in the dataset.

The overall distribution of attendance as well as its logarithmic smoothening is demonstrated in Figure 2. As seen from the distribution histograms, original variable is skewed to the left having the biggest number of matches with a very low attendance. Meanwhile, after logarithmic transformation multi-modal distribution is observed. That might be driven by heterogeneity of matches clustered according to effects that we seek to test in our model. We can hypothesize that at least four different clusters may be distinguished.

To cope with possible heteroskedasticity, we have used an estimator robust regression. Moreover, the exploratory variable attendance and one of the predictors –price- have been smoothed out by logarithmic transformation (see Figure 2).

## Results and Findings

The results are split into two groups according to the steps of the research design. The first group of the empirical tests are presented in Table 1. The specification describes a demand function which includes a part of price effect factors which point to the rationality of the uncertainty of outcome hypothesis and irrational emotional factors which refer to brand-teams and star-players.

**TABLE 1:** Descriptive statistics of the main variables.

Variable	Obs	Mean	SD	Min	Max
Attendance	1,047	4,406.24	6,416.53	32	52,989
Brand-team playing at home	1,049	0.22	0.41	0	1
Brand-team playing away	1,049	0.2	0.4	0	1
Price for ticket	1,047	21.46	11.73	3.01	91.69

Table 2 shows the outputs of four models’ estimations. As has been discussed in the research design, we gradually introduce variables of our interest. Some factors previously tested in the literature and mentioned above (i.e., competitive intensity, quality of venue, specific star players, etc.) are not included in our model due to the lack of data. However, the omission of such variables does not constitute an issue – as observed in several other demand-based papers in the literature. With a benchmark of the first basic model, which is conditioned just by parabolic function of uncertainty, we test how interaction effect with brand-team dummies might change outputs of estimations and sought coefficients.

**TABLE 2:** Results of models estimation.

Variables	(1) ln_attendance	(2) ln_attendance	(3) ln_attendance	(4) ln_attendance
ln_price	−0.0129 (0.0573)	−0.0235 (0.0558)	0.00498 (0.0570)	−0.0562 (0.0553)
Uncertainty	−1.581*** (0.604)	−1.118** (0.529)	−0.831 (0.532)	−0.846 (0.528)
Uncertainty^2^	1.242*** (0.426)	0.911** (0.386)	0.740* (0.390)	0.770** (0.382)
Uncert_Brand_h	–	1.015*** (0.200)	–	−0.217 (0.267)
Brand	1.414*** (0.149)	1.491*** (0.0964)	1.967*** (0.0957)	–
Brand_h	–	0.231** (0.104)	–	2.077*** (0.145)
Playoff	0.792*** (0.106)	0.689*** (0.103)	0.773*** (0.105)	0.547*** (0.102)
Uncert_Brand	1.113*** (0.213)	–	–	–
Uncert_Brand_a	–	–	1.285*** (0.205)	−0.770*** (0.266)
Brand_a	–	–	−0.705*** (0.109)	1.783*** (0.148)
Constant	7.740*** (0.252)	7.641*** (0.229)	7.450*** (0.229)	7.650*** (0.227)
Season FE	Yes	Yes	Yes	Yes
State FE	Yes	Yes	Yes	Yes
Observations	1,112	1,112	1,112	1,112
R-squared	0.696	0.714	0.701	0.723

Standard errors in parentheses. ***p < 0.01, **p < 0.05, *p < 0.1.

The first notable result of the estimations refers to insignificant relationship between attendance and ticket price. Recalling that we suspected price to be endogenous we find this output important. The interpretation may be driven by a real relative insignificance of price compared to the rest of drivers: uncertainty and brand-team phenomena. Ticket price in the Brazilian State Championship is relatively low, does not influence decision to attend a game, and as a result does not bring substantial variety to the explained variable of our model. However, this factor is treated in our study as a control variable.

**FIGURE 3:**
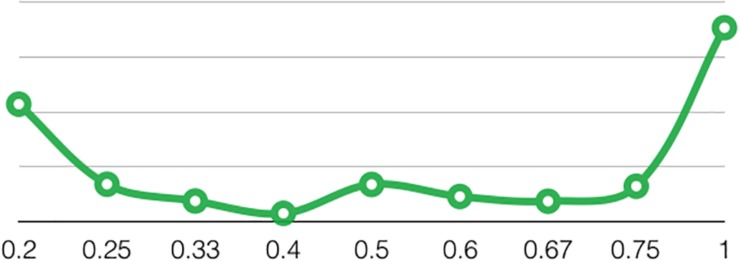
The U-shape behavior of uncertainty in Brazilian State Championship.

We have to focus on uncertainty together with brand-team interaction. We have found a U-shape behavior of uncertainty of match outcome. Taking that the metric of uncertainty is a discrete variable as measured in this research we have to look more precisely at the outputs of the model (Figure 3). The minimum value of uncertainty equals 0.39. This means that only 0.2, 0.25, and 0.33 is on the left part of the parabola, which corresponds to a very low value of attendance that is close to 2,500. As suspected, great attendance on the left and right part of the revealed parabola is driven by joint influence of two effects that we need to separate in the following models.

All moderation effects of brand-team on uncertainty are significantly positive and weaken the isolated uncertainty effect. If we compare these influences with pure brand-team effect, we can see that brand-team is relatively more important and brings greater contribution for attendance. This finding shows that emotional drivers and perceptions might be treated as a more important factor with football spectators. This result is robust, as demonstrated in all estimated models. As has been expected, this effect has to be relatively greater for the brand-team playing away. We hypothesized that spectators prefer to attend matches to take advantage of watching a strong team playing. Nevertheless, we failed to find evidence for that. On contrary, the brand-team playing at home provides a more substantial effect on match attendance according to the outputs of our estimations. This is reasonable as those teams have larger fan bases than small clubs. The overall effect is significant and positive for matches with the participation of brand-team. In other words, separating the additional effect of brand-team on uncertainty we have found out an interesting phenomenon that contradicts a classical theory of uncertainty-driven attendance – football fans prefer to attend matches with a strong team as an opponent even though a prediction of match outcome is high. This effect is stronger when the brand-team plays in its city. This happens as these matches constitute derby games (brand-team vs. brand-team) that usually drive greater emotions.

In order to understand better the effect of the participation of a brand-team in the matches, we split the sample into two subsamples. Table 3 presents the results of both subsamples. We can observe how the price turns to be significant in both and, at the same time, when there is not a brand-team in the match the sign the effect of price in attendance is negative but when there is a brand team the effect becomes positive. This is an interesting result because, in the first case, the result follows the economic logic as the demand decreased with an increase of price. At the same time, a more attractive team simultaneously creates an increase in the price and in the demand. Regarding UO, when there are not brand-teams involved, we have found a U-shape relationship. That means that if there are not brand-teams, fans prefer witnessing a secure victory of their team over a stronger opponent. However, in the case of the subsample with brand-teams, the effect of UO is linear but is compensated by the moderation effect of the presence of a brand-team as visitor. It is worth remarking that the average price is almost two times when we are talking about brand-teams in the match.

**TABLE 3:** Results of the model in the two subsamples.

Variables	Without brand-teams	With brand-teams
	(1)	(2)
	ln_attendance	ln_attendance
ln_price	−0.299***	0.578***
	(0.0704)	(0.104)
Uncertainty	−2.527***	1.118*
	(0.742)	(0.645)
Uncertainty*Brand_a	–	−1.336**
		(0.604)
Uncertainty*Brand_h	–	−0.000326
		(0.613)
Uncertainty^2^	2.044***	−0.287
	(0.538)	(0.766)
CV	Included	–
Constant	8.213***	6.652***
	(0.325)	(0.384)
Observations	644	403
R-squared	0.447	0.561
Average price	15.84	30.43
	(6.48)	(12.62)

Standard errors in parentheses. ***p < 0.01, **p < 0.05, *p < 0.1

The second group of the results refers to a price mechanism affected by emotional irrationality which might contradict conventional demand function. Looking at the regression estimated on the entire sample, we observe no significant effect of the ticket price to the match attendance. However, this can be sensitive to the subsamples which represent substantially different spectators’ behavior. We hypothesize, leaning on the previous sports psychological literature, that emotional factors may distort the decision-making process and make spectators less sensitive to price level when brand-teams play on a pitch. To test that, we split our setting to two different subsamples and estimate the same specification, excluding only not relevant controls.

As one can observe, the findings appear to draw a very interesting interpretation. We discover a normal demand function only for non-brand-team matches. Importantly, we have shown that price is negative and significant when presumably no dominance of irrational emotions takes place. However, the second model, estimated on the subsample of matches with brand-team participation, shows the price has a positive significant effect. We would avoid direct interpretation of this result addressing possible endogeneity brought by irrational factors of price mechanism which covariate with error term.

In trying to cope with this empirical problem we propose an instrumental variable with an argument that prices are set by match organizers who are aware of the emotions of spectators who demonstrate specific demand for matches with brand-teams. This reasoning allows us to propose the following variable which appears to be exogenous to a price having no direct covariation with the attendance of a match. We presume that match organizers must look at the previous attendance of similar matches in the past establishing prices for further events. Spectators, meanwhile, are unlikely to be directly influenced by the demand of similar matches in the historical period. In other words, when deciding whether it is worth attending a match, one is not interested in how many spectators have watched similar matches before. This interpretation is a sufficient condition to use attendance of the matches with the same opponents in the previous seasons to instrument price for the current season. Certainly, this solution significantly restricts our subsample.

For this subsample, we select cases matching games of the three seasons of the Brazilian State Championship with the same combination of home- and away-team playing. We instrument the ticket price by the attendance in the previous season, controlling for all possible factors. The results are shown in Table 4. As it can be observed, again the price becomes not significant and, at the same time, the moderation effect of a visiting brand-team in UO results significant and negative. Thus, a strong emotional component has been discovered again – with a brand-team visiting the fans will attend the match even when they probably expect a victory of the away-team.

**TABLE 4:** Results of the analysis with IV.

**Variables**	**With brand-teams**	**IV**
	**(1)**	**(2)**
	**ATTENDANCE_ln**	**PRICE_ln**
ln_price_hat	0.613	–
	(0.707)	
Uncertainty	0.0364	
	(1.947)	
Uncertainty*Brand_a	−2.553***	
	(0.899)	
Uncertainty*Brand_h	−0.450	
	(0.883)	
Uncertainty^2^	1.234	
	(1.978)	
CV	Included	
Lagged_ln_Attendance	–	0.204***
		(0.0267)
Constant	6.912**	1.419***
	(2.767)	(0.201)
Observations	71	180
R-squared	0.566	0.247

Standard errors in parentheses. ***p < 0.01, **p < 0.05, *p < 0.1.

## Discussion of Key Findings

This paper addressed the impact of two different sources of emotions attracting fans to live football matches: uncertainty of outcome and brand perception. We set rational factors of spectators’ behavior against emotional irrationality and studied how their balance can influence demand for football matches. Our findings show evidence that price for tickets is not important for match attendance in any specification with brand-team effect. We interpret that other drivers have substantially higher relevance than price. The fact that price is not relevant comes to corroborate the idea that fans are driven more by emotions than by economic reasoning. Looking for marginal effects of uncertainty jointly with an imbalanced game, we have discovered that the effect of brand-team playing is relatively more significant. That means that traditional models with the phenomenon of highly competitive matches do not work in the case when in Brazilian championships the brand-team is playing against a small one. Moreover, this result is robust and more significant when the brand-team is playing at home.

A peculiarity of the Brazilian State Championship is that it imposes regional opponents’ assignments. This breaks the connection between brand-team participation and high uncertainty of outcome. That happens because there is a significant number of matches when the team from the first division is playing against a team from lower divisions. The statistics during the two observed seasons say that about 47% of matches with brand-team participation were played against a relatively weak opponent, which means a very low uncertainty of outcome. The technical level of correlation is around -0.53. However, theoretical correlation does not exist. The interaction term between uncertainty of outcome and brand-team participation identifies the potential marginal effect on attendance with highly competitive matches between strong teams. Moreover, by introducing these effects by non-linear functional form, we can identify the local minimum of uncertainty for both cases (with brand-teams or just ordinary matches). This allows us to disentangle emotional and rational phenomena of attendance. Importantly, it shows that the coefficients have a significant difference in all specifications.

Clubs can use the fact of the presence of brand-teams to raise the price for tickets. In that sense, if clubs are looking for profits, fans can pay a higher pricefor the tickets. Considering the emotional behavior of the fans, the governing bodies of football should take care of them by maybe limiting maximum prices that can be charged. Sharing the revenues for tickets can help to keep the prices lower when a brand-team is playing away. With this, local fans can enjoy the presence of brand-teams in their own stadiums without a great increase of price, as the club knows that part of the revenues in the venue of the brand-team will end in its pocket. This kind of competition provides a ‘cross-subsidy’ for small clubs. This should be considered by the National Federation when considering whether to close this competition. Some other National Federations worldwide could consider the opportunity of implementing this kind of championship.

**FIGURE 4:**
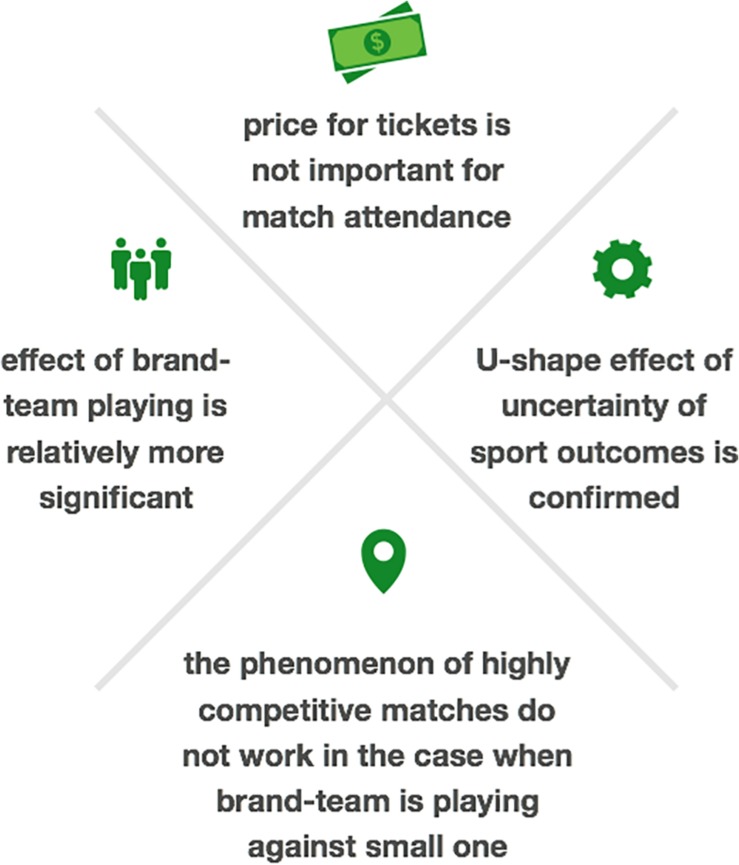
Key findings of the study.

However, when two subsamples based on a presumably higher relevance of irrational factors are created – one with brand-teams and other without brand-teams – we find that in the matches without brand-teams the effect of the price becomes negative as should be expected for normal demand curve. Meanwhile, when a brand-team is playing, the effect turns to be positive indicating significantly higher levels of endogeneity of the price when a strong team is one of the opponents. By introducing instrumental variable for the entire sample, we discovered that a price effect is not significant anymore and shows that all other included drivers of attendance pulls the variation of attendance. This finding is coherent with the literature and shows that demand for tickets in football has a very low elasticity which is in line with the idea of emotions as the main driver for attendance. A summary of the key findings is depictured on Figure 4.

## Data Availability Statement

All data used in this research are freely available and can be found at http://www.srgoool.com.br. Dataset prepared by the authors from that source is available on request.

## Author Contributions

ES, TG, and AB have contributed equally to this work.

## Conflict of Interest

The authors declare that the research was conducted in the absence of any commercial or financial relationships that could be construed as a potential conflict of interest.
